# Is he or she the main player in table tennis mixed doubles?

**DOI:** 10.1186/s13102-022-00612-0

**Published:** 2023-01-04

**Authors:** Zheng Zhou, Hui Zhang

**Affiliations:** Department of Sport Science, College of Education, Zhejiang University, Zhejiang, China

**Keywords:** Table tennis, Mixed doubles, Competition contexts, Striking groups, Performance analysis

## Abstract

**Background:**

Since mixed doubles have been set up in the table tennis competition of the 2020 Tokyo Olympic Games, coaches and players have paid increasing attention to mixed doubles matches. This study aims to compare and analyse male and female performance in the different contexts of table tennis mixed doubles as well as the impact of their performance on the probability of winning matches.

**Methods:**

100 matches between the top 30 mixed doubles were selected (based on the world rankings for 2019 to 2021) as samples. According to the stroke order of a mixed doubles match, the players are divided into four groups: male versus male (P_m–m_), male versus female (P_m–f_), female versus male (P_f–m_) and female versus female (P_f–f_). Then, new methods with concepts are proposed to analyse stroke performance by four groups of players in various competition contexts of mixed doubles.

**Results:**

(1) The stroke performance in the first four strokes was much better than that in the after four strokes (*P* < 0.05), and males performed better than female players in the first four strokes (*P* < 0.05). (2) The stroke performance of each group for winning matches was significantly better than that for losing matches (*P* < 0.01). (3) Players in each group performed better (*P* < 0.01) in the ahead and under control states than in the behind and lost control states. However, most stroke performance within the four groups was not significantly different in different states. (4) The impact of scoring rates by different groups on the winning probability of a mixed doubles match from high to low was P_m–f_ > P_f–f_ > P_m–m_ > P_f–m_. (5) In the actual competition, the percentage of female players serving first in each game is 79.64%, and the percentage of the stroke group of female players serving to female players receiving (P_f–f_) is 58.25%.

**Conclusion:**

This study considers several competition contexts to analyse the performance of male and female players in table tennis mixed doubles. We propose that the stroke performance of male versus female players is the most important factor affecting the results of mixed doubles matches. In addition, selecting the first server or first receiver in each game reasonably and analysing the stroke orders emphatically are also very important in mixed doubles.

## Background

Table tennis is a dynamic and interactive sport, and the performance of each player is influenced by that of the other player. The results of table tennis matches are affected by the performance of both players [1–4]. Doubles matches in table tennis involve two players on each pair in mixed, men’s and women’s doubles. Each player must not only cooperate with her or his partner but also pay attention to the stroke order of the opponents. Since the Rio Olympics, the International Table Tennis Federation (ITTF) and World Table Tennis (WTT) have set up mixed doubles sub events in many tournaments, making such sub events more important and influential. Therefore, table tennis associations, coaches and players in various countries have also continuously increased their investment in scientific research on mixed doubles in recent years and have achieved great success. For example, a Japanese pair won the first Olympic gold medal in the Tokyo Olympics; a Chinese pair won the gold medal of the World Table Tennis Championships in 2021; and pairs from Chinese Taipei, Chinese Hong Kong, Germany and France have also achieved excellent results in this sub event.

Most of the methods used in previous studies extend the “three-phase evaluation method” [5] from single to (mixed) doubles matches [6]. The basic principle and analysis process are the same; the two players from both sides are regarded as a whole, and the points gained and lost with each stroke are counted in turn and classified into three phases for analysis [7–9]. Then, the paradigm of the analyses is unified further. Some studies divided players’ scores and losses into serving and receiving rounds and built doubles technical and tactical models for analysis [10, 11]. This method can provide a good evaluation of the performance of players on both sides, but the only drawback is that opponents are still considered as a whole. Xiao et al. expanded the four serving and receiving rounds of both sides to eight (by dividing opponents into two players), improving the previous method, and the authors analysed a men’s doubles match [12].

Table tennis matches have obvious game temporal structure characteristics [13]. A game shall be won by the player or pair first scoring 11 points unless both players or pairs score 10 points when the game shall be won by the first player or pair subsequently gaining a lead of 2 points [14]. The accumulation and change of scores will cause fluctuations in players’ psychology, affecting stroke performance [15]. Therefore, players may have varying performances in different competition contexts, such as in different game stages and score states in each game. Almost all studies divided the game stages into three: start, middle and end game, but the specific classification boundaries are different. In the previous studies, 4 and 8 points had been made in a game, called the middle and end stages, respectively [15], and 1–4 points, after 9 points regarded as the beginning and crucial [16], and scores of 0–4, 5–8 and above 9 mark the start, middle and end stages, respectively [17], and more specifically, the start stage as occurring before a player scores 4 points, the middle stage as occurring when a player scores 4 points and the end stage as occurring after a player scores 8 points [18]. However, the division of score states has not been provided in table tennis academic research.

Mixed doubles differ from singles and men’s (women’s) doubles because players of different genders strike the ball alternately. Although the traditional three-phase evaluation method can reflect the overall strength of players in matches, it cannot show the performance characteristics and differences of male or female players in different competition contexts. In addition, to the best of our knowledge, no studies have focused on the performance of male and female players in mixed doubles.

Therefore, this study aims to analyse the stroke performance of players in mixed doubles and proposes the following hypotheses: (a) male and female players perform differently in different contexts; (b) the impact of their performance on winning probability is different.

## Methods

### Match samples

This study selected 100 matches between the top 30 mixed doubles rankings (based on the world rankings of 2019 to 2021). There were 33 pairs involved, including 18 pairs ranked 1–10, 8 pairs ranked 11–20, and 7 pairs ranked 21–30. Both pairs were analysed in each match [data taken from the ITTF (https://www.ittf.com/rankings/) and WTT (https://worldtabletennis.com/rankings). The information about the 100 matches is shown in Table 1. In addition, 14,130 points (scores and losses) from all mixed doubles pairs were analysed as raw data.

All match videos were taken from television relays or the internet. The local institutional ethics committee approved the study.

**Table 1:** The information about the 100 matches

Type of tournaments	N	Year of tournaments	N	The best of 7/5 games	N	Level of draws	N	Identification of players	N
World Tour Open	67	2019	50	3–0	44	1/8 finals	36	European versus Asian	56
Olympic Games	14	2021	27	3–1	25	1/4 finals	32	Asian versus Asian	27
WTT Contender Series	9	2020	23	3–2	17	Semi finals	19	European versus European	11
World Tour Grand Finals	5			4–0	6	Finals	11	African versus Asian	2
World Championship	4			4–1	4	1/16 finals	2	European versus African	1
Asian Championship	1			4–3	3			Oceanian versus Asian	1
				4–2	1			Oceanian versus European	1
								North American versus Asian	1

### Performance indicators and data collection

#### Classification of striking groups in mixed doubles

According to the order of play in table tennis doubles, the server shall do service, and the receiver shall then make a receive, the partner of the server shall then make a return, and the partner of the receiver shall then make a return. After that, each player in turn in that sequence shall make a return [14]. At the end of a rally, there are only two results, namely, score or loss [19].

Therefore, in mixed doubles, the results of matches can be summarized as the strokes performance of players in four groups (Table 2), which include male versus male, male versus female, female versus male and female versus female groups. Dividing players into four groups and analysing strokes can help verify whether there are differences in performance between males and females in mixed doubles.

**Table 2:** Classification of striking groups and performance indicators in mixed doubles

Groups	Performance indicators	Results
Male player versus male player (P_m–m_)	Stroke: first (serve), second (receive), third, fourth, fifth…, and last stroke	Score/lose
Male player versus female player (P_m–f_)		
Female player versus male player (P_f–m_)		
Female player versus female player (P_f–f_)		

#### Data collection system

A table tennis data collection system was used for stroke information collection in this study [20–22]. The objectivity of the observation indicators was confirmed through the agreement of two independent observers using Cohen’s kappa statistics (inter-rater agreement) [23]. Five matches were selected from the examined games for this purpose. Cohen’s kappa values (k) of the observation indicators were found to be valued at k = 1 for the “strike number” and for “scoring or losing”.

### The models of score states and game stages

A scoring system of “points–games–match” was adopted in a table tennis match, and different matches often contain different numbers of games, such as the best of 7 or 5 games. In this study, two pairs (each pair has a male player and a female player) play against each other, namely, P_A_ and P_B_ in mixed doubles. For game g, we denote the rally scores of pairs P_A_ and P_B_ as RS′_A_ (g) and RS′_B_ (g), respectively. We denote ${\text{RS}}_{{\text{A}}}^{\prime } \left( {\text{g}} \right) + {\text{RS}}_{{\text{B}}}^{\prime } \left( {\text{g}} \right)$ as ${\text{RS}}_{{{\text{Sum}}}}^{\prime } \left( {\text{g}} \right)$ and $\left| {{\text{RS}}_{{\text{A}}}^{\prime } \left( {\text{g}} \right) - {\text{RS}}_{{\text{B}}}^{\prime } \left( {\text{g}} \right)} \right|$ as ${\text{RS}}_{{{\text{Dif}}}}^{\prime } \left( {\text{g}} \right)$.

There are many combinations of rally scores in a game of table tennis matches. In this study, we define 6 score states: normal glued, key glued, ahead, behind, under control and lost control in each game for the following reasons: (a) in table tennis, each rally starts with a serve (2 serves alternating between each player, and 1 serve when the score reaches 10–10), and the opponent receives until scored by one of them [24] and (b) the scores that occur closer to the end of the game have more significant impacts on the game’s outcome [25]. (c) A gap of 3–4 points only needs to win one serving and receiving turn by the player, while a difference of more than 5 points needs several serving and receiving turns, which is very difficult.

The six score states S (s) are as follows:

$${\text{S }}\left( {\text{s}} \right) = \left\{ {\begin{array}{*{20}c} {{\text{normal}}\;{\text{glued,}}} \\ {{\text{key}}\;{\text{glued,}}} \\ {{\text{ahead,}}\;{\text{behind,}}} \\ {{\text{under,}}\;{\text{lost}}\;{\text{control,}}} \\ \end{array} } \right.{\text{}}\begin{array}{*{20}c} {{\text{Max}}\left( {{\text{RS}^{\prime}}_{{\text{A}}} \left( {\text{g}} \right),{\text{RS}^{\prime}}_{{\text{B}}} \left( {\text{g}} \right)} \right) \le 9,{\text{RS}^{\prime}}_{{{\text{Sum}}}} \left( {\text{g}} \right) \le 16,{\text{RS}^{\prime}}_{{{\text{Dif}}}} \left( {\text{g}} \right) \le 2} \\ {{\text{Min}}\left( {{\text{RS}^{\prime}}_{{\text{A}}} \left( {\text{g}} \right),{\text{RS}^{\prime}}_{{\text{B}}} \left( {\text{g}} \right)} \right) \ge 8,{\text{RS}^{\prime}}_{{{\text{Sum}}}} \left( {\text{g}} \right) > 16,{\text{RS}^{\prime}}_{{{\text{Dif}}}} \left( {\text{g}} \right) \le 2} \\ {3 \le {\text{RS}^{\prime}}_{{{\text{Dif}}}} \left( {\text{g}} \right) \le 4} \\ {{\text{RS}^{\prime}}_{{{\text{Dif}}}} \left( {\text{g}} \right) \ge 5} \\ \end{array}$$

**Fig. 1:**
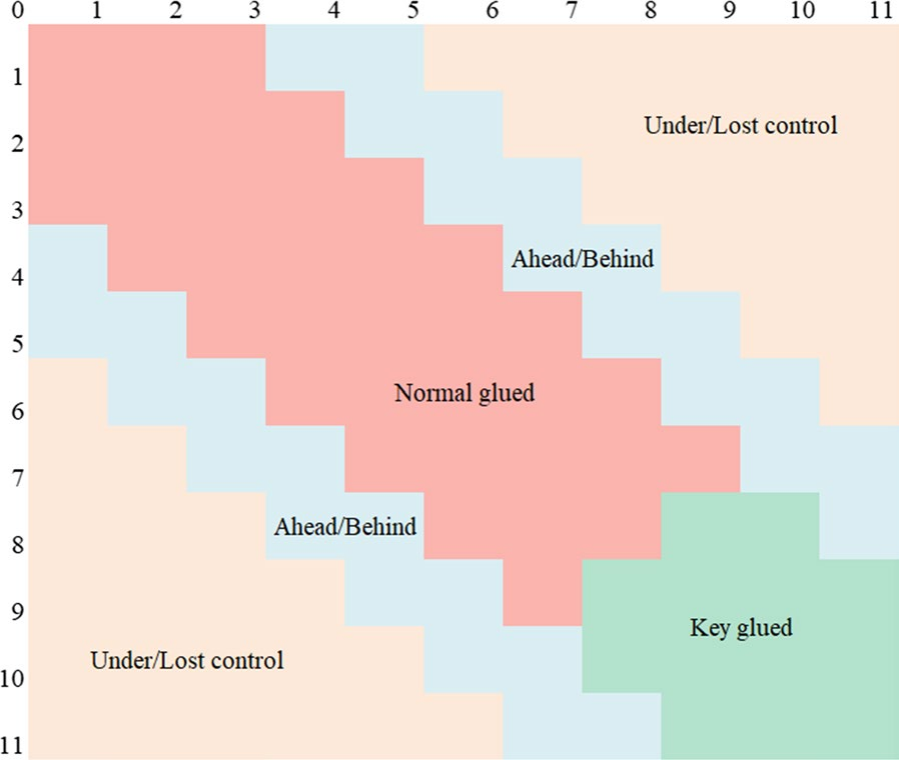
The attribution of rally scores in normal/key glued, ahead/behind and under/lost control states. Note: The key glued state in green also includes scores above 11, which are not shown in Fig. 1 for simplicity, such as 11:12, 16:16, and 20:19

The attribution of rally scores in different score states is shown in Fig. 1.

According to previous studies on the classification of game stages [15–18], we define three-game stages: start, middle and end.

The game stages G (s) are as follows:

$${\text{G }}\left( {\text{s}} \right) = \left\{ {\begin{array}{*{20}c} {{\text{start,}}} \\ {{\text{middle,}}} \\ {{\text{end,}}} \\ \end{array} } \right.\begin{array}{*{20}c} {\text{Max}}\left( {{\text{RS}}_{{\text{A}}}^{\prime } \left( {\text{g}} \right),{\text{RS}}_{{\text{B}}}^{\prime } \left( {\text{g}} \right)} \right) \le 4 & {} \\ 4<{\text{Max}}\left( {{\text{RS}}_{{\text{A}}}^{\prime } \left( {\text{g}} \right),{\text{RS}}_{{\text{B}}}^{\prime } \left( {\text{g}} \right)} \right) \le 8 & {} \\ {\text{Max}}\left( {{\text{RS}}^{\prime } _{{\text{A}}} \left( {\text{g}} \right),{\text{RS}}^{\prime } _{{\text{B}}} \left( {\text{g}} \right)} \right) > 8 & {} \\ \end{array}$$

**Fig. 2:**
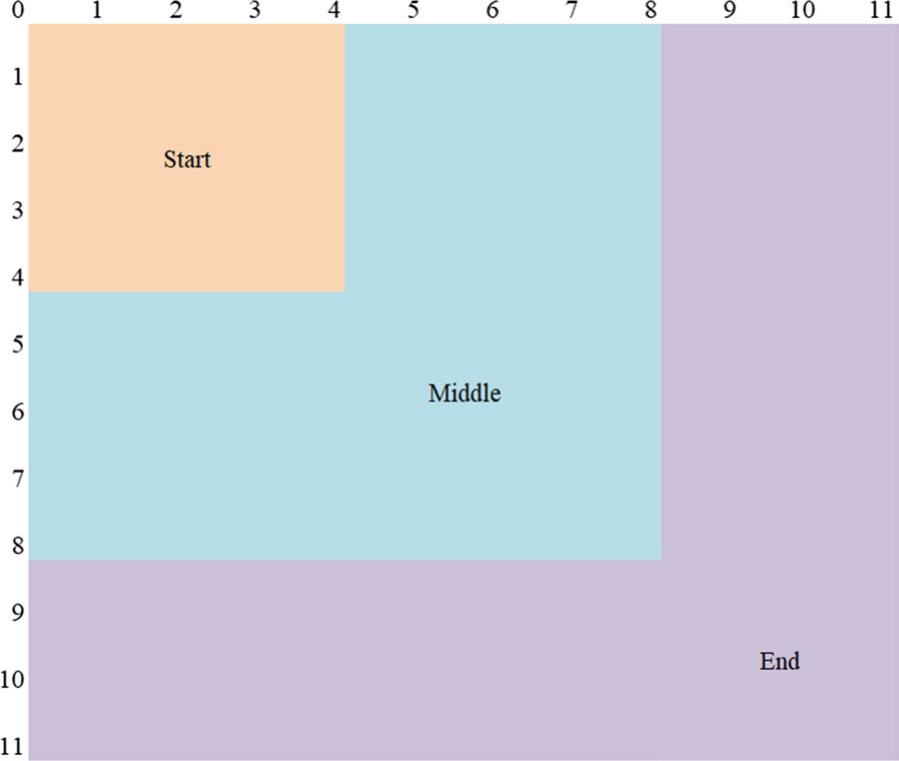
The attribution of rally scores in the start, middle and end game stages. Note: In the end game stage, it also includes the scores after 11 on both sides, such as 11:12, 16:16 and 20:19

The attribution of rally scores in different game stages is shown in Fig. 2.

### Computation of the scoring rate (SR), the losing rate (LR) and stroke effectiveness (SE)

We adopt a concept and algorithm for calculating the stroke scoring rate and losing rate and effectively analysed the stroke performance of players in single matches [26]. In mixed doubles matches, four players strike the ball in sequence alternately, which means that each player’s second strike comes after the other three players’ strokes. Sometimes, a certain rally result has little or nothing to do with a player’s stroke. Therefore, to better analyse the performance of each player, the method based on the number of strokes is the most suitable for mixed double matches.

Let s_i_ be the rally number of the ith strokes scored, let l_i_ be the rally number of the ith strokes lost, let N_i_ be the rally number of the ith strokes, let SR_i_ (Eq. 1) be the scoring rate of the ith strokes, let LR_i_ (Eq. 2) be the losing rate of the ith strokes, and let SE_i_ (Eq. 3) be the effectiveness of the ith strokes. SR, LR and SE are computed by the following Eq. 

1 $${\text{S}\text{R}}_{\text{i}}={\text{s}}_{\text{i}}/{\text{N}}_{\text{i}}$$

2 $${\text{L}\text{R}}_{\text{i}}={\text{l}}_{\text{i}}/{\text{N}}_{\text{i}}$$

3 $$\text{S}{{\text{E}}_{\text{i}}=\text{S}\text{R}}_{\text{i}}-{\text{L}\text{R}}_{\text{i}}$$

The scoring rate (SR), losing rate (LR) and stroke effectiveness (SE) is defined as follows [26]: The scoring rate (SR) represents how good the scoring strikes are at the ith strokes. The losing rate (LR) represents the poor stability or receiving strikes at the ith strokes. The LR will be low if a player has good defensive strikes or stability. Stroke effectiveness (SE) represents the scoring or losing tendency at the ith stroke. Even if a player has good offensive strikes and a high SR value, the value of SE can be low when the player’s stroke is liable to fail, and LR has a high value. SE can be regarded as the contribution of the ith strokes to winning a match.

### Regression and path analysis of scoring rate and win probabilities

In table tennis mixed doubles, the stroke scoring rate of male and female players facing opponents of different genders has an impact on the outcome of matches. Therefore, this study determines the influence of each group’s scoring rate on the probability of winning matches through regression equations. On this basis, a path analysis model of table tennis mixed doubles is constructed to reveal the direct and indirect effects of the scoring rate for the four groups.

### Statistical analysis

All statistical tests were performed using SPSS version 24.0 software (SPSS Inc., Chicago, IL, USA) for Windows, and statistical significance was established at *P* < 0.05. The effect size of the T-test was estimated by Cohen’s *d* [27], interpreted as small (0.20), medium (0.50) or large (0.80), and the effect size of the F-test was estimated by squared association indices [28], interpreted as small (0.04), medium (0.25) or large (0.64).

## Results

This chapter introduces the SR, LR and SE of strokes of the four groups (P_m–m_, P_m–f_, P_f–m_ and P_f–f_) in several respects.

### Stroke features in the first four strokes and after four strokes

Table 3 shows that the SR and SE of each group for the first four strokes were larger than that of after four strokes, and the LR of each group for the first four strokes was smaller than that of after four strokes, and their differences are significant [except for the SR of group P_f–m_ (*P* = 0.043), all other *P* values < 0.01]. This reveals that in a mixed doubles match, the impact of the first four strokes on the result of the match is significantly greater than that of after four strokes.

**Table 3:** SR, LR and SE values of each group for the first four strokes and after four strokes

	N	First four strokes	After four strokes	t	*P*	d
P_m–m_						
SR	100	0.399 ± 0.085	0.350 ± 0.136**	3.044	0.003	0.430
LR	100	0.294 ± 0.065	0.570 ± 0.149***	− 16.946	< 0.001	2.396
SE	100	0.106 ± 0.098	− 0.219 ± 0.265***	11.477	< 0.001	1.622
P_m–f_						
SR	100	0.402 ± 0.076	0.332 ± 0.133***	4.527	< 0.001	0.640
LR	100	0.300 ± 0.083	0.601 ± 0.152***	− 17.366	< 0.001	2.456
SE	100	0.102 ± 0.121	− 0.267 ± 0.273***	12.359	< 0.001	1.748
P_f–m_						
SR	100	0.377 ± 0.081	0.341 ± 0.159*	2.040	0.043	0.289
LR	100	0.328 ± 0.087	0.583 ± 0.179***	− 12.875	< 0.001	1.821
SE	100	0.049 ± 0.137	− 0.242 ± 0.325***	8.265	< 0.001	1.169
P_f–f_						
SR	100	0.366 ± 0.075	0.324 ± 0.109**	3.227	0.001	0.456
LR	100	0.309 ± 0.072	0.594 ± 0.119***	− 20.431	< 0.001	2.889
SE	100	0.057 ± 0.111	− 0.269 ± 0.204***	14.115	< 0.001	1.996
Total						
SR	400	0.386 ± 0.081	0.337 ± 0.135***	6.266	< 0.001	0.443
LR	400	0.308 ± 0.078	0.587 ± 0.151***	− 32.844	< 0.001	2.323
SE	400	0.079 ± 0.120	− 0.249 ± 0.270***	22.209	< 0.001	1.571

(1) The evaluation criteria of d are as follows: small effect (0.20 ≤ d < 0.50); medium effect (0.50 ≤ d < 0.80); and large effect (d ≥ 0.80). (2) *P < 0.05, **P < 0.01, and ***P < 0.001

**Fig. 3:**
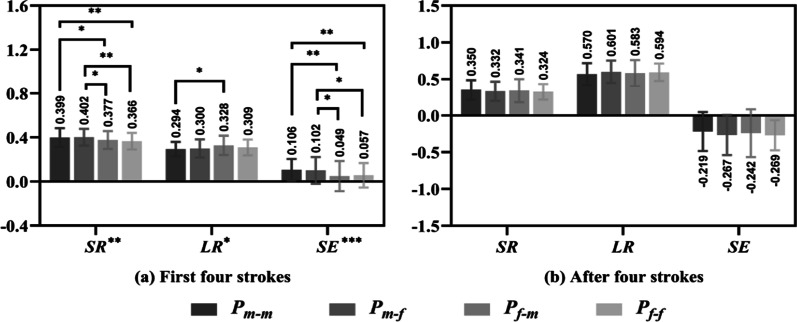
SR, LR and SE of the four groups in the first four strokes and after four strokes

Figure 3 compares the SR, LR and SE values of the four groups for the first four strokes and after four strokes. In the first four strokes (Fig. 3a), the SR and SE of groups P_m–m_ and P_m–f_ were significantly higher than those of groups P_f–m_ and P_f–f_. In contrast, the LR of groups P_m–m_ and P_m–f_ were lower than those of groups P_f–m_ and P_f–f_, but only the difference between groups P_m–m_ and P_f–m_ was significant (*P* < 0.05). However, in the after four strokes, there was no significant difference among the four groups (Fig. 3b). The results show that in the first four strokes of mixed doubles, male players have higher SR and SE values and lower LR values than female players.

### Comparison of stroke features across different match results

Table 4 shows that the SR and SE of each group for winning matches were larger than those for losing matches, the LR of each group for winning matches was smaller than that for losing matches, and their differences were significant (all *P* < 0.01). It is worth noting that the SE of each group among the losing matches was negative.

**Fig. 4:**
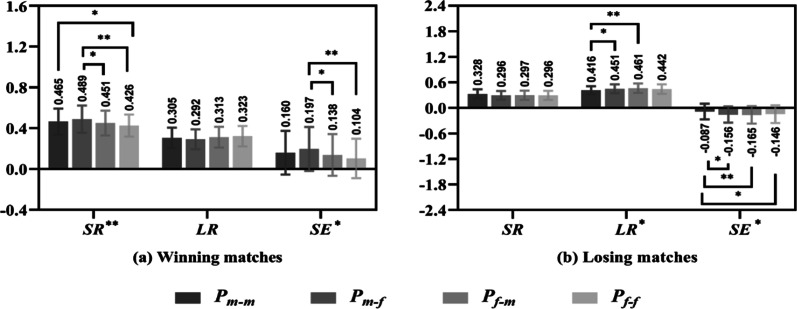
SR, LR and SE of four groups among winning and losing matches

Figure 4 compares the SR, LR and SE values of the four groups for the winning and losing matches. For the winning matches (Fig. 4a), group P_m–f_ had the highest SR (0.489) and SE (0.197) values and the lowest LR (0.292) value. Group P_f–f_ had the highest LR (0.323) value and lowest SR (0.426) and SE (0.104) values. The SR and SE of group P_m–f_ were significantly greater than those of groups P_f–m_ and P_f–f_ (P_m–f_ versus P_f–m_: both *P* < 0.05; P_m–f_ versus P_f–f_: both *P* < 0.01), and the SR of group P_m–m_ was significantly higher than that of group P_f–f_ (*P* < 0.05). However, no significant difference was found in LR values among the four groups. The results suggest that the SR and SE of group P_m–f_ play an important role in mixed doubles.

**Table 4:** SR, LR and SE of each group among winning and losing matches

	N	Winning	Losing	t	*P*	d
P_m–m_						
SR	100	0.465 ± 0.128	0.328 ± 0.108***	8.168	< 0.001	1.155
LR	100	0.305 ± 0.100	0.416 ± 0.093***	− 8.088	< 0.001	1.144
SE	100	0.160 ± 0.214	− 0.087 ± 0.184***	8.747	< 0.001	1.237
P_m–f_						
SR	100	0.489 ± 0.134	0.296 ± 0.101***	11.506	< 0.001	1.627
LR	100	0.292 ± 0.097	0.451 ± 0.104***	− 11.195	< 0.001	1.583
SE	100	0.197 ± 0.215	− 0.156 ± 0.189***	12.308	< 0.001	1.741
P_f–m_						
SR	100	0.451 ± 0.121	0.297 ± 0.109***	9.448	< 0.001	1.336
LR	100	0.313 ± 0.102	0.461 ± 0.111***	− 9.837	< 0.001	1.391
SE	100	0.138 ± 0.204	− 0.165 ± 0.205***	10.468	< 0.001	1.480
P_f–f_						
SR	100	0.426 ± 0.108	0.296 ± 0.107***	8.551	< 0.001	1.209
LR	100	0.323 ± 0.100	0.442 ± 0.112***	− 7.896	< 0.001	1.117
SE	100	0.104 ± 0.194	− 0.146 ± 0.207***	8.778	< 0.001	1.241
Total						
SR	400	0.458 ± 0.125	0.304 ± 0.107***	18.679	< 0.001	1.321
LR	400	0.308 ± 0.100	0.442 ± 0.106***	− 18.383	< 0.001	1.300
SE	400	0.150 ± 0.209	− 0.138 ± 0.198***	19.989	< 0.001	1.414

In the lost matches (Fig. 4b), the LR of group P_m–m_ was significantly lower than that of groups P_m–f_ and P_f–m_ (*P* < 0.05 and *P* < 0.01, respectively). In addition, there were significant differences in SE values between groups P_m–m_ and P_m–f_, P_f–m_, P_f–f_ (*P* < 0.05, *P* < 0.01 and *P* < 0.05, respectively). However, there was no significant difference in SR values among the four groups.

### Comparison of stroke features under different score states

There is only one result in each rally of table tennis matches in which one side wins or loses a point (the other side wins), and each point contributes or is lost to the match’s outcome to different degrees. Therefore, it can help coaches to find the performance differences between players clearly and intuitively by comparing the performance of players under the same score differences, even the same score differences between the end of a game and other moments.

#### The normal and key glued states

Table 5 shows the SR, LR and SE of each group for the normal and key glued states. The comparison of the two states shows that only group P_f–m_ had significant differences in SR and SE values (both *P* < 0.05), and the SR and SE of group P_f–m_ in the normal glued state were greater than those in the key glued state.

**Fig. 5:**
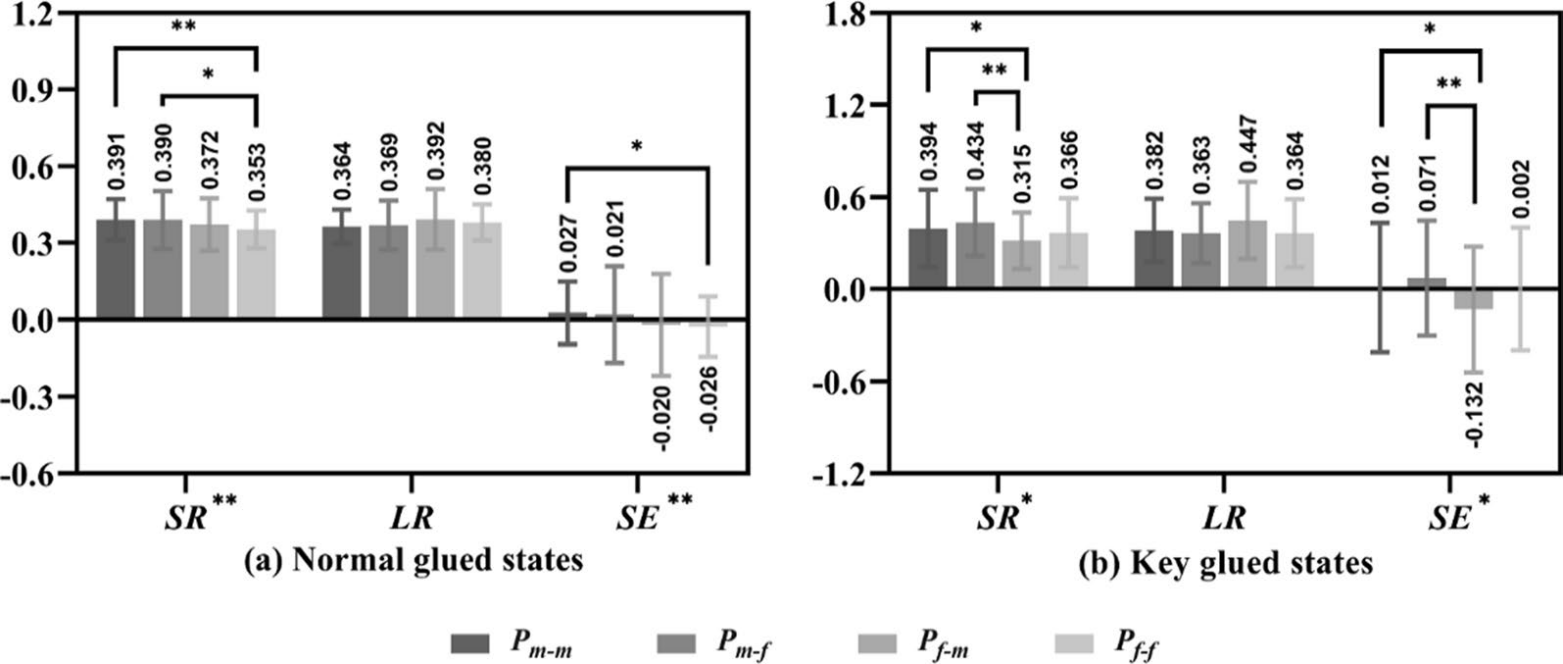
SR, LR and SE of the four groups in the normal and key glued states

The performance of the four groups in the two states is shown in Fig. 5. In the normal glued states (Fig. 5a), groups P_m–m_ and P_m–f_ had relatively high SR (0.391 and 0.390, respectively) and SE (0.027 and 0.021, respectively). However, both the P_f–m_ and P_f–f_ groups had negative SE values of − 0.020 and − 0.026, respectively. Among them, there were significant differences in SR and SE values between groups P_m–m_ and P_f–f_ (*P* < 0.01, *P* < 0.05), and there was a significant difference in SR values between groups P_m–f_ and P_f–f_ (*P* < 0.05).

In the key glued states (Fig. 5b), group P_m–f_ shows the highest SR (0.434) and SE (0.071) values, and group P_f–m_ presents the lowest SR (0.315) and SE (− 0.132) values. There were significant differences between group P_m–f_ and group P_f–m_ (both *P* < 0.01). Group P_m–m_ presented greater SR (0.394) and SE (0.012) values and was significantly different from group P_f–m_ (both *P* < 0.05).

**Table 5:** SR, LR and SE of each group for the normal and key glued states

	N_1_/N_2_	Normal glued	Key glued	t	*P*	d
P_m–m_						
SR	100/78	0.391 ± 0.080	0.394 ± 0.251	− 0.092	0.927	0.014
LR	100/78	0.364 ± 0.067	0.382 ± 0.206	− 0.756	0.452	0.120
SE	100/78	0.027 ± 0.122	0.012 ± 0.420	0.301	0.764	0.048
P_m–f_						
SR	100/68	0.390 ± 0.113	0.434 ± 0.218	− 1.507	0.135	0.249
LR	100/68	0.369 ± 0.096	0.363 ± 0.195	0.233	0.816	0.039
SE	100/68	0.021 ± 0.188	0.071 ± 0.375	− 1.008	0.316	0.167
P_f–m_						
SR	100/63	0.372 ± 0.103	0.315 ± 0.183*	2.231	0.028	0.379
LR	100/63	0.392 ± 0.119	0.447 ± 0.252	− 1.628	0.107	0.280
SE	100/63	− 0.020 ± 0.199	− 0.132 ± 0.408*	2.031	0.046	0.349
P_f–f_						
SR	100/75	0.353 ± 0.073	0.366 ± 0.225	− 0.454	0.651	4.291
LR	100/75	0.380 ± 0.070	0.364 ± 0.223	0.592	0.555	4.506
SE	100/75	− 0.026 ± 0.117	0.002 ± 0.399	− 0.589	0.557	0.095
Total						
SR	400/284	0.377 ± 0.095	0.379 ± 0.225	− 0.131	0.896	0.011
LR	400/284	0.376 ± 0.091	0.387 ± 0.220	− 0.800	0.425	0.065
SE	400/284	0.001 ± 0.162	− 0.009 ± 0.405	0.353	0.724	0.029

#### The ahead, behind, under control and lost control states

**Fig. 6:**
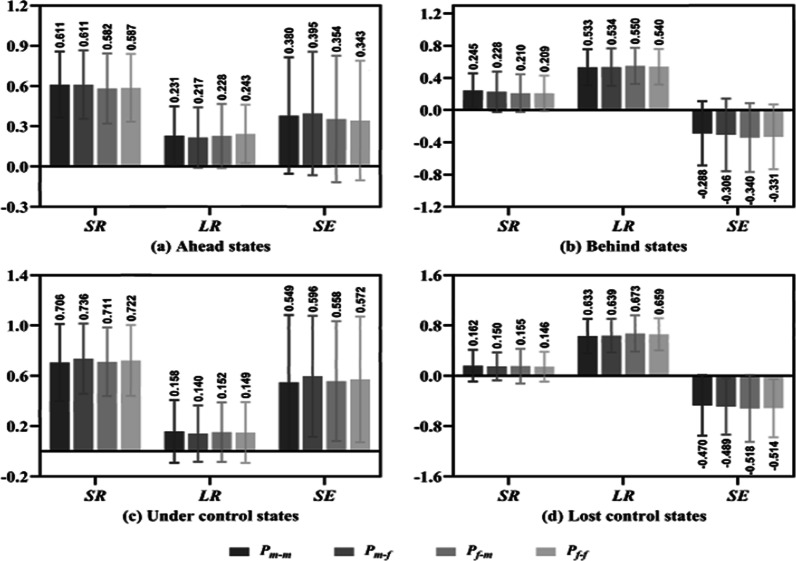
SR, LR and SE of four groups for the ahead, behind, under control and lost control states

Tables 6 and 7 show the performance of each group in the ahead and behind states and for the under and lost control states, respectively. Among them, the SR, LR and SE of each group show significant differences between the ahead and behind states as well as between under and lost control states (all *P* < 0.01). Differences between the four groups were analysed under each state, as shown in Fig. 6. In the ahead state (Fig. 6a), group P_m–f_ performed best on SR (0.611), LR (0.217) and SE (0.395) values, and group P_f–m_ performed worst on SR and SE values, which reached 0.582 and 0.354, respectively. For the behind states (Fig. 6b), each group played poorly, all SR and SE were lower than 0.3 and − 0.2, respectively, and all LR were higher than 0.5.

**Table 6:** SR, LR and SE of each group for ahead and behind states

	N_1_/N_2_	Ahead	Behind	t	*P*	d
P_m–m_						
SR	97/97	0.611 ± 0.246	0.245 ± 0.212***	11.091	< 0.001	1.593
LR	97/97	0.231 ± 0.218	0.533 ± 0.223***	− 9.557	< 0.001	1.373
SE	97/97	0.380 ± 0.434	− 0.288 ± 0.399***	11.153	< 0.001	1.601
P_m–f_						
SR	97/99	0.611 ± 0.255	0.228 ± 0.252***	10.578	< 0.001	1.511
LR	97/99	0.217 ± 0.225	0.534 ± 0.231***	− 9.756	< 0.001	1.394
SE	97/99	0.395 ± 0.460	− 0.306 ± 0.451***	10.766	< 0.001	1.538
P_f–m_						
SR	96/94	0.582 ± 0.261	0.210 ± 0.235***	10.311	< 0.001	1.497
LR	96/94	0.228 ± 0.239	0.550 ± 0.223***	− 9.620	< 0.001	1.396
SE	96/94	0.354 ± 0.471	− 0.340 ± 0.428***	10.623	< 0.001	1.542
P_f–f_						
SR	97/98	0.587 ± 0.253	0.209 ± 0.219***	11.144	< 0.001	1.595
LR	97/98	0.243 ± 0.218	0.540 ± 0.220***	− 9.451	< 0.001	1.354
SE	97/98	0.343 ± 0.446	− 0.331 ± 0.402***	11.083	< 0.001	1.587
Total						
SR	387/388	0.598 ± 0.253	0.223 ± 0.230***	21.565	< 0.001	1.550
LR	387/388	0.230 ± 0.225	0.539 ± 0.223***	− 19.251	< 0.001	1.383
SE	387/388	0.368 ± 0.452	− 0.316 ± 0.419***	21.840	< 0.001	1.569

**Table 7:** SR, LR and SE of each group for under- and lost control states

	N_1_/N_2_	Under control	Lost control	t	*P*	d
P_m–m_						
SR	81/83	0.706 ± 0.307	0.162 ± 0.252***	12.400	< 0.001	1.939
LR	81/83	0.158 ± 0.249	0.633 ± 0.273***	− 11.648	< 0.001	1.820
SE	81/83	0.549 ± 0.534	− 0.470 ± 0.482***	12.834	< 0.001	2.003
P_m–f_						
SR	82/81	0.736 ± 0.279	0.150 ± 0.221***	14.844	< 0.001	2.324
LR	82/81	0.140 ± 0.224	0.639 ± 0.266***	− 12.949	< 0.001	2.030
SE	82/81	0.596 ± 0.481	− 0.489 ± 0.447***	14.904	< 0.001	2.335
P_f–m_						
SR	77/78	0.711 ± 0.273	0.155 ± 0.276***	12.602	< 0.001	2.024
LR	77/78	0.152 ± 0.237	0.673 ± 0.288***	− 12.296	< 0.001	1.977
SE	77/78	0.558 ± 0.476	− 0.518 ± 0.532***	13.273	< 0.001	2.133
P_f–f_						
SR	82/79	0.722 ± 0.283	0.146 ± 0.237***	14.010	< 0.001	2.025
LR	82/79	0.149 ± 0.242	0.659 ± 0.255***	− 13.038	< 0.001	2.055
SE	82/79	0.572 ± 0.499	− 0.514 ± 0.463***	14.302	< 0.001	2.256
Total						
SR	322/321	0.719 ± 0.285	0.153 ± 0.246***	26.934	< 0.001	2.124
LR	322/321	0.150 ± 0.237	0.651 ± 0.270***	− 25.019	< 0.001	1.974
SE	322/321	0.570 ± 0.496	− 0.497 ± 0.480***	27.698	< 0.001	2.185

For the under control states (Fig. 6c), male players playing against female players always had relative advantages over other groups; for example, the SR and SE of group P_m–f_ were 0.736 and 0.596, respectively, and the LR (0.140) was the lowest. For the lost control states (Fig. 6d), the performance of each group was worse than that of the behind states; all SR were lower than 0.2, LR were higher than 0.6 and SE were lower than 0.4. However, there was no significant difference between the four groups (*P* > 0.05) in the ahead, behind, under control or lost control states.

### Comparison of stroke features in different game stages

**Fig. 7:**
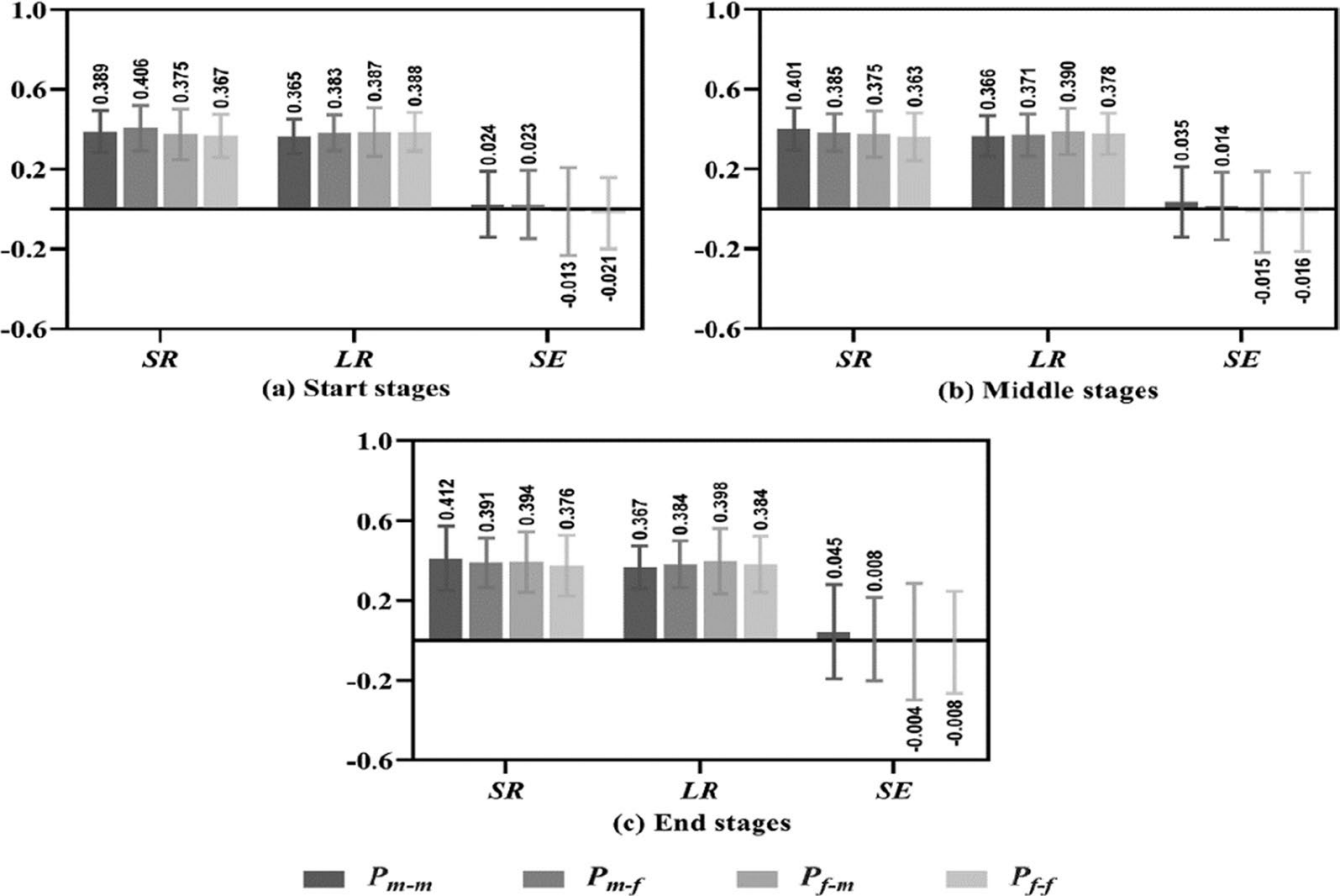
SR, LR and SE of the four groups in the start, middle and end stages

Table 8 shows each group’s SR, LR and SE for the start, middle and end stages. There was no significant difference (*P* > 0.05) in the SR values of each group between the three-game stages or in the LR and SE values. However, the SR and SE of each group in the end stage were higher than those in the start and middle stages (except the SR and SE of group P_m–f_ in the end stage were lower than those in the start stage). In addition, there was no significant difference (*P* > 0.05) between the four groups across the start, middle and end stages (Fig. 7)

**Table 8:** SR, LR and SE of each group for the start, middle and end stages

	N	Start	Middle	End	F	*P*	η^2^
P_m–m_							
SR	100	0.389 ± 0.105	0.401 ± 0.106	0.412 ± 0.162	0.751	0.473	0.005
LR	100	0.365 ± 0.087	0.366 ± 0.101	0.367 ± 0.108	0.009	0.991	0.001
SE	100	0.024 ± 0.165	0.035 ± 0.176	0.045 ± 0.236	0.282	0.755	0.002
P_m–f_							
SR	100	0.406 ± 0.113	0.385 ± 0.093	0.391 ± 0.123	1.030	0.359	0.006
LR	100	0.383 ± 0.089	0.371 ± 0.105	0.384 ± 0.117	0.466	0.628	0.003
SE	100	0.023 ± 0.171	0.014 ± 0.169	0.008 ± 0.209	0.172	0.842	0.001
P_f–m_							
SR	100	0.375 ± 0.127	0.375 ± 0.116	0.394 ± 0.152	0.684	0.505	0.005
LR	100	0.387 ± 0.122	0.390 ± 0.116	0.398 ± 0.164	0.128	0.880	0.001
SE	100	− 0.013 ± 0.220	− 0.015 ± 0.202	− 0.004 ± 0.292	0.052	0.949	0.001
P_f–f_							
SR	100	0.367 ± 0.108	0.363 ± 0.119	0.376 ± 0.153	0.210	0.811	0.002
LR	100	0.388 ± 0.096	0.378 ± 0.103	0.384 ± 0.141	0.235	0.791	0.001
SE	100	− 0.021 ± 0.179	− 0.016 ± 0.197	− 0.008 ± 0.256	0.085	0.918	0.001
Total							
SR	400	0.384 ± 0.114	0.381 ± 0.110	0.393 ± 0.148	0.210	0.811	0.002
LR	400	0.381 ± 0.099	0.376 ± 0.106	0.383 ± 0.134	0.235	0.791	0.001
SE	400	0.004 ± 0.185	0.005 ± 0.187	0.010 ± 0.250	0.085	0.918	0.001

### The relationship between the scoring rate and win probabilities

The multiple R, R squared and adjusted R squared of the regression model exceed 0.9, and the scoring rate of the four groups explains 91.2% of the winning probability, showing that the model presents a good fit. The variance analysis results show that the regression equation is significant (F = 506.925, *P* < 0.01). Table 9 shows the regression analysis results of the model coefficients. The minimum value of tolerance is 0.755, and all VIF are less than 2. Each independent variable of the equation has a significant effect on the dependent variable (*P* < 0.01) according to the T test. Therefore, an equation is established, and the regression model of mixed doubles is Y = 0.071 + 0.260 × _1_ + 0.306 × _2_ + 0.239 × _3_ + 0.324 × _4_. According to unstandardized coefficients, the impact of scoring rates of male and female players against opponents on the probability of winning matches is ranked from high to low: X_2_ (P_m–f_) > X_4_ (P_f–f_) > X_1_ (P_m–m_) > X_3_ (P_f–m_).

**Table 9:** Results of the regression model coefficient of mixed doubles

Model	Unstandardized coefficients		Standardized coefficients beta	t	*P*	Collinearity-statistics	
	B	SE				Tolerances	VIF
Constant	0.071	0.010		7.109	< 0.001		
X_1_ (P_m–m_)	0.260	0.020	0.308	12.940	< 0.001	0.795	1.258
X_2_ (P_m–f_)	0.306	0.018	0.404	17.096	< 0.001	0.807	1.239
X_3_ (P_f–m_)	0.239	0.020	0.286	11.724	< 0.001	0.755	1.324
X_4_ (P_f–f_)	0.324	0.021	0.352	15.078	< 0.001	0.824	1.213

**Fig. 8:**
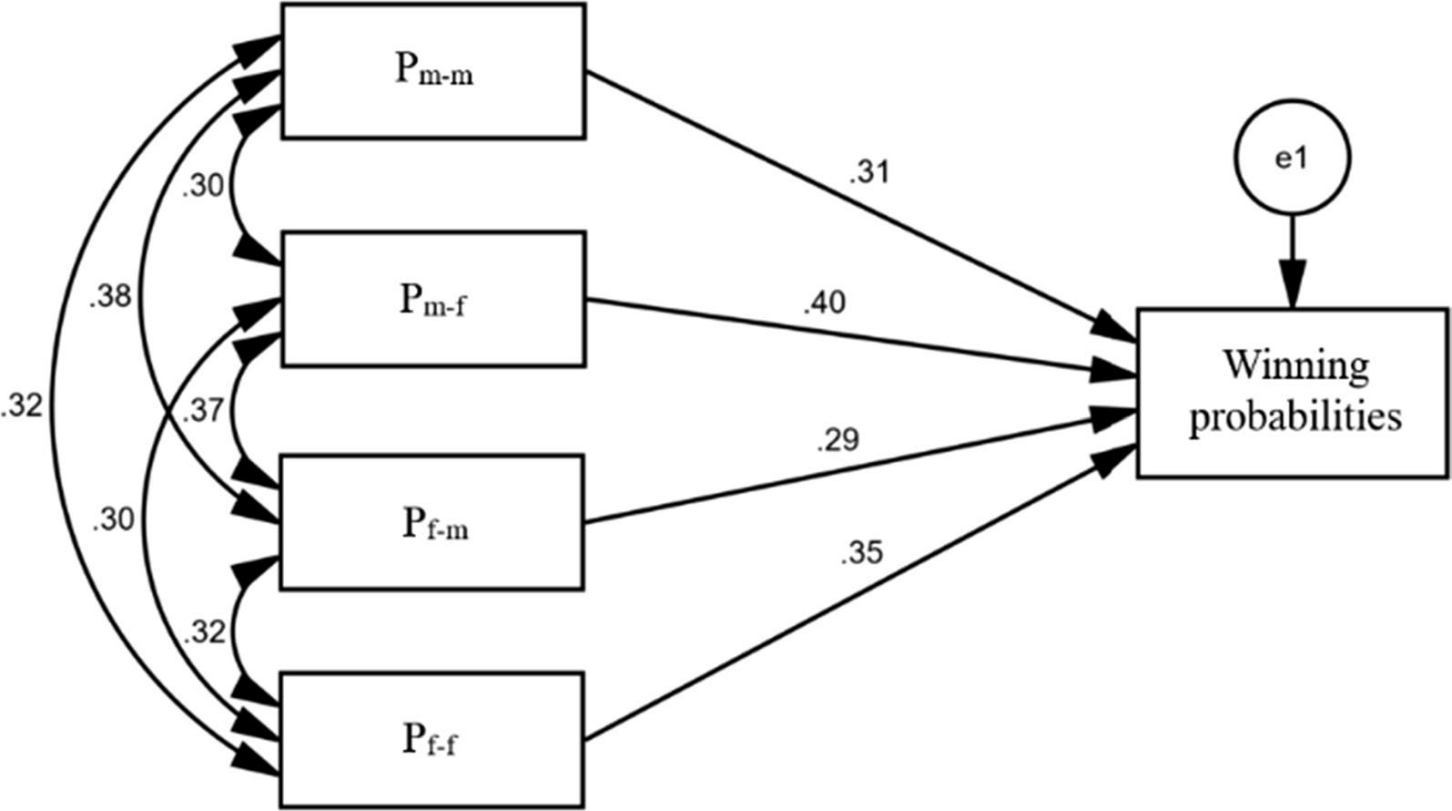
Path analysis model of table tennis mixed doubles

Figure 8 shows the relationships between four independent variables (groups P_m–m_, P_m–f_, P_f–m_ and P_f–f_) and one dependent variable (winning probabilities of matches) in the path analysis model. There are significant correlations between the scoring rates of the four groups as well as with the winning probabilities of matches (*P* < 0.001). Table 10 shows the path coefficients of the mixed doubles matches. Among them, variable X_2_ (P_m–f_) presents the largest direct path coefficient (0.404) and smallest indirect path coefficient (0.305), followed by variable X_4_ (P_f–f_), whose direct and indirect path coefficients are 0.352 and 0.314, respectively. In contrast, variables X_3_ (P_f–m_) and X_1_ (P_m–m_) present larger indirect path coefficients (0.383 and 0.342) and lower direct path coefficients (0.286 and 0.308). In addition, the order of the total determined coefficient from large to small is X_2_ (0.286) > X_4_ (0.234) > X_1_ (0.200) > X_3_ (0.191), which is the same as the order of the unstandardized coefficients.

**Table 10:** The path coefficient (PC) of the mixed doubles model

Variable	Total effect	Direct PC	Indirect PC	→X_1_	→X_2_	→X_3_	→X_4_	TDC
X_1_ (P_m–m_)	0.650	0.308	0.342		0.120	0.110	0.113	0.200
X_2_ (P_m–f_)	0.709	0.404	0.305	0.091		0.107	0.107	0.286
X_3_ (P_f–m_)	0.669	0.286	0.383	0.118	0.151		0.114	0.191
X_4_ (P_f–f_)	0.666	0.352	0.314	0.099	0.122	0.093		0.234
Total	–	–	–	0.307	0.393	0.310	0.334	0.912

→X_1_, X_2_, X_3_ and X_4_ indicate the indirect path coefficients of the independent variables through X_1_, X_2_, X_3_ and X_4_, respectively

TDC denotes the total determined coefficient

## Discussion

This study aims to compare the stroke performance of male and female players in different contexts of table tennis mixed doubles and the impact of their performance on the outcome of matches.

### The first and after four strokes, and the order of strokes

The results in Table 3 reveal that male and female players performed significantly better in the first four strokes than those in the after four strokes. Male players performed much better than female players regardless of the gender of the opponent in the first four strokes but performed similarly in the after four strokes (Fig. 3). It seems to indicate that male and female players competed more intensely for the first four strokes in mixed doubles, due to most of changes (including stroke speed, strength, rotation, etc.) occur in the first four strokes [29].

The results in Table 11 show that the percentage of serving first by female players (79.64%) was much higher than that by male players (20.36%). The percentage (58.25%) of “female serve to female receive” (P_f–f_) was also higher than that of the other three groups. Before a mixed doubles match began, the referee will determine the first server and the first receiver. To avoid the formation of a stroke order in which an opponent male player strikes the ball to own female player (P_m–f_), the players on one side who have the right to serve will choose to let the female player serve first. Similarly, the other side will also choose to let the female player receive first, which can prevent the opponent male player from striking the ball to the own female player in the third stroke and form an advantageous situation in which the own male player strikes the ball to the opponent female player in the fourth stroke.

**Table 11:** The information on the serve and receive in each game by male and female players

Game	Serve															
	1st		2nd		3th		4th		5th		6-7th		Total		Ratio	
Receive	M	F	M	F	M	F	M	F	M	F	M	F	M	F	M	F
Male	17	26	9	15	13	21	5	13	2	6	2	2	48	83	12.37%	21.39%
Female	1	56	12	64	6	60	8	30	4	13	0	3	31	226	7.99%	58.25%
Total	100		100		100		56		25		7		388		100%	

Therefore, it can help coaches and players understand the importance and nature of stroke orders in mixed doubles. It can also help them focus on training in the first four strokes, especially in the round that the opponent female serves and the own female player receives.

### Performance differences of players in different match results, score states and game stages

The results show that male players performed significantly better than female players on SR and SE values in the winning matches (Fig. 4a), in the normal and key glued states (Fig. 5). However, there was no difference on performance between male and female players in the LR values of winning matches, in the SR values of losing matches (Fig. 4), in the ahead, behind, under control and lost control states and in the start, middle and end stages (Figs. 6, 7).

It seems to indicate the following: (a) dividing different rally scores by score states may better reflect the performance differences of male and female players in mixed doubles than doing so according to game stages; (b) the rules of table tennis mixed doubles (where four players strike the ball in turns, which is unlike badminton and tennis doubles, where one player can strike the ball consecutively) make the process of competition fairer and the outcomes more uncertain.

Therefore, coaches and players can realize further that the key to winning mixed doubles matches may lie in cooperation, complementarity and the balancing of the strengths of male and female players on a pair rather than in the outstanding performance of one player.

### The scoring rate and winning probabilities

To prove the impact of performance by male and female players on the probability of winning the game is different, this study uses the scoring rate as the performance indicator for the following reasons:

1. The results of the effective equation can be positive or negative, which is equal to the difference between the scoring and losing rates. In addition, for results obtained by a quadratic calculation, where values of scoring and losing rates are in a black box, coaches and players cannot achieve the most intuitive stroke performance.
2. In mixed doubles, a player with a lower losing rate may not have a higher scoring rate. For example, the losing rate of a female player against a female player (P_f-f_) is not the lowest in the after four strokes, but the former’s scoring rate is lower than that of the other three groups (Table 3). In contrast, the higher the scoring rate is, the higher the probability of winning a match.

In addition, the results in Table 10 show that the SR of group P_m–f_ will indirectly cause the other three groups to have a positive effect on winning probabilities and is not easily affected by other groups, followed by group P_f–f_. The total determined coefficient is the product of the correlation coefficient and direct path coefficient, indicating the total influence of each independent variable on the dependent variable in various ways. Therefore, the scoring rate of male players against female players has the greatest influence on the probability of winning matches, followed by that of female players against female players.

## Limitations of the proposed methods

The proposed methods do not consider the specific technical and tactical variables or other aspects to minimally justify the comparison. At the same time, it provides some information about the performance difference between male or female players when facing opponents of different genders in the same standard (score or loss) and the impact of scoring rates on winning probabilities. However, the previous study proposed that there are a large number of tactical types combined with nine stroke techniques and nine stroke placements each; for example, the tactics (stroke_1→2→3_) in the receiving round by male players had 999 tactical types in 225 singles matches, which are shown in Table 12 [30].

Therefore, if the specific behaviours of each stroke by four players with random stroke order need to be labelled, this method will make the data too scattered to find characteristics and differences and require many professional persons and time to collect data.

**Table 12:** Basic data of all tactics of table tennis matches [30]

Match type	Serve round			Receive round		
	Sum	Tactic type (Stroke_1→2→3_)	Mean usage rate	Sum	Tactic type (Stroke_2→3→4_)	Mean usage rate
Male						
RH_A_ versus RH_B_	8633	303	0.33%	6280	999	0.10%
RH versus LH	2683	218	0.46%	1889	653	0.15%
LH versus RH	2612	210	0.48%	1894	644	0.16%
LH_A_ versus LH_B_	1072	171	0.58%	788	394	0.25%
Female						
RH_A_ versus RH_B_	4770	403	0.25%	3423	719	0.14%
RH versus LH	1436	191	0.52%	924	355	0.28%
LH versus RH	1424	176	0.57%	990	419	0.24%
LH_A_ versus LH_B_	643	112	0.89%	477	194	0.52%

In Table 12, mean usage rate = (1/tactic type) × 100%. For instance, in the present study, the number of tactics for matches between right-handed male players in the serving round was 8633, which was 303 tactic types, and the mean usage rate was 0.33%, meaning that every tactic was used 28.49 times on average

## Conclusion

This study considers several competition contexts to analyse the performance of male and female players in table tennis mixed doubles. The results show that due to a rule requiring four players take turns striking the ball, there is no significant difference in stroke performance between male and female players in most competition contexts (e.g., in the after four strokes; in the ahead, behind, under control or lost control states; and in the start, middle or end stages). However, this study also shows that male players perform significantly better than female players in certain cases (e.g., within the first four strokes, in winning and losing matches and in the normal and key glued states), and that group male players competing against female players has the greater impact on the outcomes of mixed doubles matches. In addition, selecting the first server or first receiver in each game reasonably and analysing the stroke orders emphatically are very important in mixed doubles.

## Data Availability

The datasets used and/or analysed during the current study are available from the corresponding author on reasonable request.

## Abbreviations

P_m-m_: Male player vs. male player
P_m-f_: Male player vs. female player
P_f-m_: Female player vs. male player
P_f-f_: Female player vs. female player
SR: Scoring rate
LR: Losing rate
SE: Stroke effectiveness
VIF: Variance inflation factor
PC: Path coefficient
TDC: Total determined coefficient
